# Clinico-Demographic Profiles of Herpes Zoster Cases in Patients With and Without COVID-19 Infection During the Pandemic: A Retrospective Analysis of 32 Cases

**DOI:** 10.7759/cureus.40063

**Published:** 2023-06-06

**Authors:** Ghazal Ahmed, Satyaki Ganguly, Jemshi S Rahim, Anju George C, Habib Md R Karim

**Affiliations:** 1 Dermatology, Venereology and Leprosy, All India Institute of Medical Sciences, Deoghar, Deoghar, IND; 2 Dermatology, Venereology and Leprosy, All India Institute of Medical Sciences, Raipur, Raipur, IND; 3 Anesthesiology, Critical Care, and Pain Medicine, All India Institute of Medical Sciences, Raipur, Raipur, IND

**Keywords:** clinical characteristics, hospital epidemiology, herpes zoster reactivation, varicella-zoster, covid-2019

## Abstract

Background: Coronavirus disease 2019 (COVID-19) has multiple impacts on the human body. The immunological effect is one of the prominent ones, which is thought to be fundamental in many physical manifestations and disease severity. Herpes zoster (HZ) reactivation has been well-linked to immunity; immunocompromised states predispose a person to HZ. Studies have raised concerns about HZ incidences in COVID-19 patients; however, the clinical characteristics of the HZ cases among patients with and without COVID-19 are another area to be explored.

Methods: In this retrospective analysis, we compared the clinical and demographic characteristics of HZ cases presented to our outpatient department immediately before and during the early second wave of the COVID-19 pandemic (September 2020 to April 2021) in India. The cases were divided into two groups based on the history of COVID-19 infections. The clinico-demographic characteristics were then compared using an unpaired t-test, Fisher’s exact test, and analysis of variance as applicable using InStat software; a two-sided p-value <0.05 was considered significant.

Results: During the period, 32 cases (17 HZ cases with a history of COVID-19; 15 HZ cases without) were detected. The age and gender distribution were indifferent statistically. Our analysis showed that multi-dermatomal and disseminated involvements were significantly higher in HZ cases having a history of COVID-19.

Conclusion: The present retrospective analysis of 32 cases indicates that persons who suffered from COVID-19 and presented with HZ were likely to have a higher chance of multi-dermatomal and disseminated involvement. While our analysis cannot establish a true association between COVID-19 infection and HZ reactivation, which will require a large-scale study, clinicians might get a clue of the possible progression of the extent of HZ manifestations from our findings.

## Introduction

Coronavirus disease 2019 (COVID-19) has racked human lives. The causative agent of COVID-19 has been proven to cause immune dysregulation in the infected person [1]. The reactivation of varicella-zoster virus in dorsal root ganglia causing herpes zoster (HZ) has been reported in immunocompromised patients with malignancies, human immunodeficiency virus infection, organ transplantations, long-term immunosuppressive therapy, etc. [2]. It has been recently reported in COVID-19 patients as well. However, whether the reactivated HZ is different in clinical characteristics in those with COVID-19 is unknown. In the present study, we evaluated the history and clinical findings of 32 cases to characterize HZ in post-COVID-19 patients and compare them with those without a known infection of COVID-19.

## Materials and methods

Study setting and design

The present analysis was performed at a tertiary care academic institute (All India Institute of Medical Sciences, Raipur) in India. The outpatient services for non-COVID-19 cases were resumed with a limited number of patients, along with a dedicated service for COVID-19. Furthermore, telemedicine service was also provided during that time. Informed verbal consent was obtained from the patients for the possible use of clinical data, and informed written consent was obtained for the clinical images. However, as the analysis was not pre-planned as a case series, no formal approvals from the institutional research cell and ethical committee were sought. Nevertheless, the authors have not intervened in the management, nor have any additional procedures or investigations been done. The data were analyzed retrospectively.

Participants

Adult patients of either male or female gender attending the outpatient department from September 2020 to April 2021 were included in the analysis. Only those patients directly examined by the authors were included, and teleconsultation and review consultation data were not analyzed except for noting the progression of lesions and pain. We planned the current analysis with convenient sampling, and entire data for eligible participants were included.

Study definition

A patient was categorized to have suffered from COVID-19 if he or she was diagnosed through either rapid antigen or polymerase chain reaction tests. Patients who did not require organ and oxygen support were categorized as mild, those who required facemask oxygenation or high-flow nasal cannula oxygenation as moderate, and those requiring organ support along with non-invasive or invasive mechanical ventilation were categorized as severe COVID-19.

Data collection and outcome variables

Patients' age, gender, history of COVID-19 and mode of diagnosis, treatment, vaccination for COVID-19, and comorbidities were noted. HZ-related data, namely, dermatomes involved, types of lesions, body area, and laterality of involvements, were noted. The patient was asked to rate the pain on a 0-10 scale when the patient could understand, and then the pain score was categorized as mild, moderate, and severe. Those who could not or did not score were asked to express the pain on a mild, moderate, and severe scale.

Statistical analysis

The master chart was prepared in Microsoft Excel 2007 (Microsoft, Redmond, WA). Categorical variables were expressed in absolute number and percentage scales. Quantitative continuous data were analyzed using an unpaired t-test. The groups were compared using the chi-square test of independence. InStat software (GraphPad Software Inc., San Diego, CA) was used for statistical tests. A two-tailed p-value of <0.05 was considered significant.

## Results

A total of 13,140 outpatient and referral footfalls were noted during the period; 4845 cases were new in the OPD, and 1703 were indoor referrals. Data from 32 HZ patients were eligible for analysis; 17 HZ cases suffered from COVID-19, and 15 HZ cases did not. The data were mainly from males aged 17-80 years. The chief complaint of the patients was pain, mild-moderate in intensity, along with skin lesions. Although the mean age of the HZ patients with a history of COVID-19 was lower than those without a history of COVID-19, the difference was statistically insignificant. Most of the patients with HZ who had COVID-19 suffered from mild COVID-19. Compared to non-COVID-19 patients, patients who had COVID-19 had relatively higher comorbidities (relative risk 1.481; 95% confidence interval 0.79-2.77, Fisher’s exact test). However, there were no statistical differences among the groups' comorbidities; the two-tailed p-value was 0.291. The distribution of COVID-19 vaccination and HZ cases was similar in both groups. The demographic data, gender distribution, details of COVID-19 infection, vaccination, and treatment received for COVID-19 are presented in Table 1.

**Table 1:** Demographic and COVID-19-related details of the cohorts COVID-19: coronavirus disease 2019; HIV: human immunodeficiency virus; NA: not applicable *Values are presented as median (minimum-maximum).

Parameters	COVID-19, yes (N=17)	COVID-19, no (N=15)
Age (mean ± standard deviation), years	49.35 ± 16.64	52.0 ± 17.98
Male, n (%)	11 (64.7)	9 (60.0)
Female, n (%)	06 (35.5)	6 (40.0)
Days since recovery from COVID-19*	75 (7-425)	NA
Severity of COVID-19: mild	13 (76.5)	NA
Severity of COVID-19: moderate	3 (17.6)	NA
Severity of COVID-19: severe	1 (5.9)	NA
COVID-19 vaccination, single dose	1 (5.9)	0
COVID-19 vaccination, two dosages	3 (7.6)	4 (26.7)
COVID-19 vaccination, none	13 (76.5)	11 (73.3)
Diabetes mellitus/HIV/steroid therapy	8 (47.1)	4 (26.7)

The onset of zoster lesions in post-COVID-19 patients was within 7-425 days, with a median of 75 days. Zoster lesion onset in post-vaccinated cases was within 9-180 days. The most common area affected in both groups was the head and neck, followed by thoracic dermatomes. Post-COVID-19 HZ had significantly higher multi-dermatomal disease compared to non-COVID-19 patients. All non-COVID-19 zoster patients had unilateral disease, whereas three (17.7%) of the post-COVID-19 zoster patients had bilateral disease, and two (11.76%) presented with disseminated lesions. Most (71%) post-COVID-19 patients took more than five days for lesions to crust compared to 41% of non-COVID-19 patients. The HZ lesion characteristics and pain scores are presented in Table 2.

**Table 2:** Clinical profiles of HZ cases of the cohort divided based on the history of COVID-19 infection COVID-19: coronavirus disease 2019; NRS: Numerical Rating Scale; HZ: herpes zoster *Some patients had multiple lesion types, mostly in the COVID-19 group.

Clinical characteristics	COVID-19, yes (N=17)	COVID-19, no (N=15)	Chi-square statistic	p-value
Region involved: head and neck	7 (41.2)	8 (53.3)	2.093	0.553
Region involved: thoracic	4 (23.5)	4 (26.7)		
Region involved: abdominal	4 (23.5)	3 (20.0)		
Region involved: disseminated	2 (11.8)	0		
Dermatomes involved, single	3 (17.6)	10 (66.7)	8.563	0.035
Dermatomes involved, 2	7 (41.2)	3 (20.0)		
Dermatomes involved, 3	5 (29.4)	2 (13.3)		
Dermatomes involved, >3	2 (11.8)	0		
Skin lesions: unilateral	14 (82.3)	15 (100.0)	4.628	0.098
Skin lesions: bilateral	3 (17.7)	0		
Disseminated and bilateral	2 (5.9)	0		
Predominant lesions: vesicle^*^	14 (82.3)	12 (80.0)	1.368	0.713
Predominant lesions: papules^*^	1 (5.9)	0		
Predominant lesions: bullous^*^	1 (5.9)	1 (6.7)		
Predominant lesions: crusted^*^	1 (5.9)	2 (13.3)		
Pain: mild (NRS 0-3)	9 (52.9)	9 (60.0)	1.573	0.455
Pain: moderate (NRS 4-6)	8 (47.1)	5 (33.33)		
Pain: could not assess	0	1 (6.67)		

Furthermore, Grade IV post-herpetic neuralgia existed in 47% of COVID-19 arm patients and 29% of non-COVID-19 arm patients. One post-COVID HZ case presented with facial palsy, whereas two patients in the non-COVID-19 arm had secondarily infected zoster lesions. All patients responded well to the oral anti-viral (acyclovir or valacyclovir). None of the patients had severe or excruciating pain, which was well controlled with pregabalin and paracetamol as assessed during the subsequent one or two visits. Antibiotics were prescribed for secondary infected cases.

**Figure 1:**
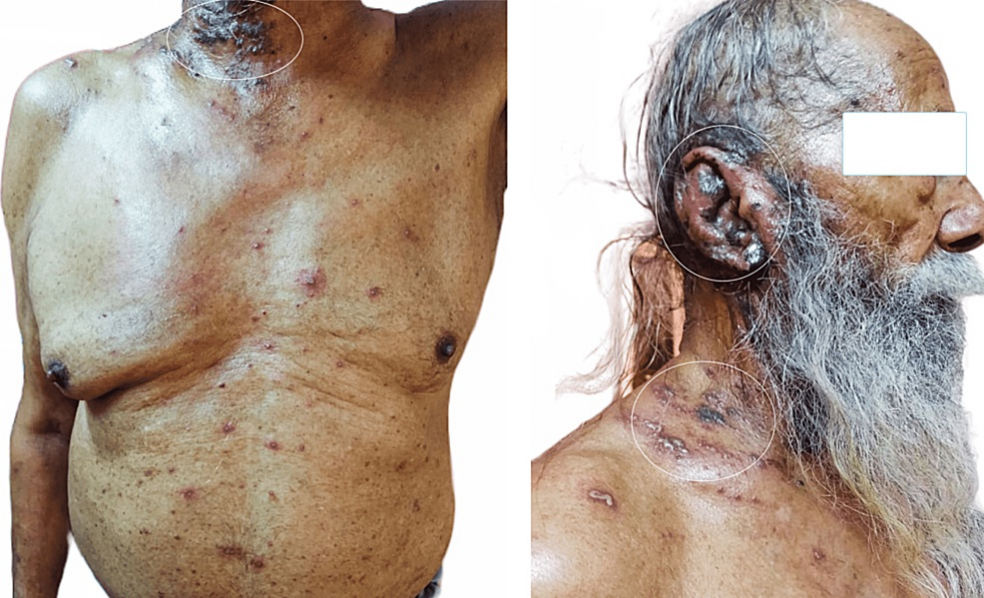
Disseminated herpes zoster infection in an elderly patient shows multiple clustered vesicles over the earlobe, neck, and supraclavicular region. The figure also shows numerous isolated lesions over the chest and abdominal wall.

Both the patients who had disseminated HZ were elderly. Figure 1 shows disseminated HZ in an 80-year-old gentleman. The elderly patient complained of painful lesions predominantly over the earlobe and neck. Lesions were also present over the thoracic and abdominal area and had bilateral distribution. On examination, vesicles over the erythematous base were present over the V3, C2, C3, and T2 dermatomes, along with more than 25 vesicles outside the involved dermatome.

## Discussion

In our case series, post-COVID-19 HZ patients had significantly more multi-dermatomal, disseminated diseases; lumbar involvement and atypical lesions were also noted. The clinical course also indicated longer healing time. These patients were comparatively younger but had more comorbidities when compared to non-COVID-19 patients. Therefore, bilateral zoster presentations and multi-dermatomal involvement might point toward the underlying COVID-19-related immune dysregulation and other immunocompromised states. Multi-dermatomal involvement was also reported in a study from Kuwait [3]. The same study also reported the HZ cases following COVID-19 vaccination.

Interestingly, four out of 15 patients in the non-COVID-19 group had been vaccinated for COVID-19, and all had HZ within three months of the vaccination, even before getting the second dose. Transient lymphocytopenia had been implicated in the same [3,4]. Voisin et al. reported a case of disseminated HZ in a hospitalized COVID-19 patient [5]. Our patients with disseminated presentations were, however, not hospitalized patients. HZ has also been indicated as a feature of latent COVID-19 [6]. Reports of HZ in severe COVID-19 patients requiring critical care management are also there, where most of the HZ cases were reported within four to seven days of hospitalization [7]. However, our patients were OPD patients and reported a wide range of time gaps between the COVID-19 infection and HZ. The literature indicates that COVID-19-induced stress and immune dysregulation predisposes such patients to HZ [8]. The wide gap between COVID-19 infection and HZ noted in our cases might be explained by the immune dysregulation after COVID-19 is prolonged, even reported for up to eight months [1].

A review article analyzing 14 articles enrolling 29 cases of HZ in COVID-19 patients could not strongly associate COVID-19 with the increased incidence of HZ in such patients [9]. However, an increased number of studies and opinions expressed in the review and other case reports indicate the same. Our case series did not intend to analyze the relationship but the differences in the clinico-demographic spectrum of HZ cases during the pandemic concerning the history of COVID-19. However, although our case series analysis has a relatively good number of cases, we relied on the history of COVID-19 infection confirmed by multiple types of laboratory testing. Some patients might have been infected, remained asymptomatic, or not tested; we did not analyze the antibodies to categorize them exactly.

Bhavsar et al. found that COVID-19 patients aged ≥50 years were at a significantly higher risk of HZ [10]. Nearly half of the patients in our cohort with HZ with a history of COVID-19 had an age of ≥50 years. Interestingly, in our cohort, the disseminated disease was noted in elderly patients only. Therefore, HZ vaccination in situations like the COVID-19 pandemic can probably be considered in immunocompromised and elderly individuals. The literature indicates an impairment of the tight junctions, a semi-permeable barrier system for mucosal integrity, in the early COVID-19 infection. Furthermore, activated immune systems and cytokine storms are well-known phenomena in moderate and severe COVID-19 [11]. Nonetheless, immunobiology is well connected to HZ infection, where T-cells are crucial in limiting the disease severity and preventing reactivation [12]. These pathophysiologies might have played some role in the more severe presentation of the HZ cases in the COVID-19 arm of our cohort. The multi-dermatomal lesions and disseminated involvement were also noted in immunocompromised HIV-infected patients compared to non-HIV patients by Vora et al. [13]. Our similar findings in patients with a history of COVID-19 also point toward the possible disarranged immunobiology caused by the coronavirus.

At the time of presentation in the OPD, the earliest presentation was on the seventh day from the onset of the HZ lesion in the non-COVID-19 arm of the cohort, while for herpetic neuralgia, it ranged from 60 to 102 days. The exact duration of the HZ illness on presentation could not be ascertained in all cases, but it was observed that many patients presented late, probably because of limited availability and use of healthcare facilities for non-COVID-19 illnesses during the pandemic. Otherwise, our cohort's outpatient prevalence rate of HZ, complications noted, and age distributions were similar to the historical cases presented in the literature [14]. However, the lesions were predominantly located in our cohort's head and neck dermatome compared to the thoracic dermatome noted in the review of 3124 HZ clinical cases by Patki et al. [14].

Our observation of cases, however, has limitations. We used convenient sampling; data were from mostly one-point contact and cases had minimal follow-up. The COVID-19 infection history was relied on as per the patients’ history. It was not verified from a database or by enquiring the test report, which might have caused the inclusion of asymptomatic COVID-19-infected patients in this group. However, seeing the shattering and overburdened healthcare system during the pandemic and the retrospective nature of the study, performing serology for every case would not have been feasible to rule out these possibilities. We also relied on the history for the presence or absence of other auto-immune diseases. One patient had HZ approximately after 425 days of COVID-19 infection, which might not have any relation to the COVID-19 infection. Furthermore, our case reports can only generate a hypothesis as we are yet to compare data from the pre-COVID-19 period, and the data is also from a minimal number of patients. Nevertheless, although the records for HZ cases were kept prospectively, we might have missed HZ cases that might have an impact on the results, which must be considered while accepting the interpretation.

## Conclusions

Herpes zoster in patients with a history of COVID-19 infection has significantly more multi-dermatomal involvement and relatively higher bilateral presentations. The disseminated and severe form of the disease was observed in the elderly, raising suspicion of increasing age as a risk factor. Although there were increased numbers of cases with immunosuppression in the HZ with COVID-19 group, the clinico-demographic presentations were otherwise not significantly different. More extensive data analysis with larger samples in the future will provide a better insight.
